# Supplementary material for: Strong polar vortex favoured intense Northern European storminess in February 2022
Source: Commun Earth Environ. 2025 Mar 27;6(1):226. doi: 10.1038/s43247-025-02175-7 (PMC11949832; doi:10.1038/s43247-025-02175-7)
Supplement: Supplementary file 2 — Supplementary Information [file 43247_2025_2175_MOESM2_ESM.pdf]

Strong polar vortex favoured intense Northern European storminess in February 2022

Ryan S. Williams[1,2*], Amanda C. Maycock[1], Vincent Charnay[1,3], Jeff Knight[4] and Inna Polichtchouk[5]

[1]School of Earth and Environment, University of Leeds, Leeds, UK

[2]British Antarctic Survey, Cambridge, UK

[3]Antarctic Research Centre, Victoria University of Wellington, New Zealand

[4]Met Office Hadley Centre, Exeter, UK

[5]European Centre for Medium-Range Weather Forecasts (ECMWF), Reading, UK

*Corresponding author: rywill@bas.ac.uk

Williams et al. – Supplementary Information

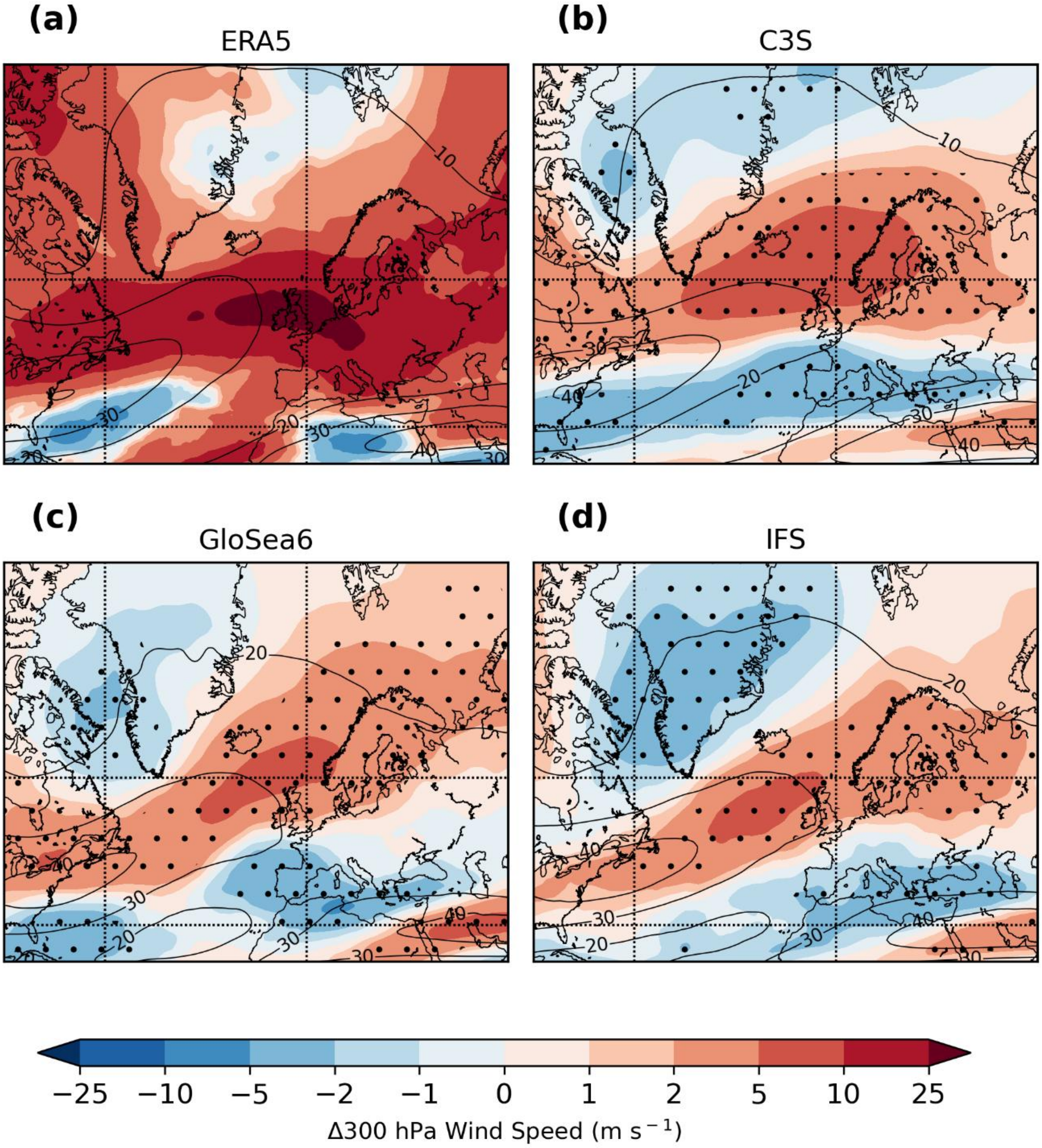

Fig. S1: Intensified upper-level North Atlantic jet stream due to the strong SPV. ***a)*** *February 2022 monthly mean 300 hPa wind speed anomaly (shading) and long-term climatology (1979-2021; contours) in ERA5.* ***b)*** *Monthly Mean difference between C3S Strong and Average SPV members (shading) and absolute wind speed according to the ensemble-mean of Average SPV members (contours).* ***c)*** *As in b) but for GloSea6, and* ***d)*** *as in c) but for IFS. Note the non-linear colour scale (units in m s^{-1}). Stippling denotes statistical significance at the 95% confidence level using a paired Student's t-test.*

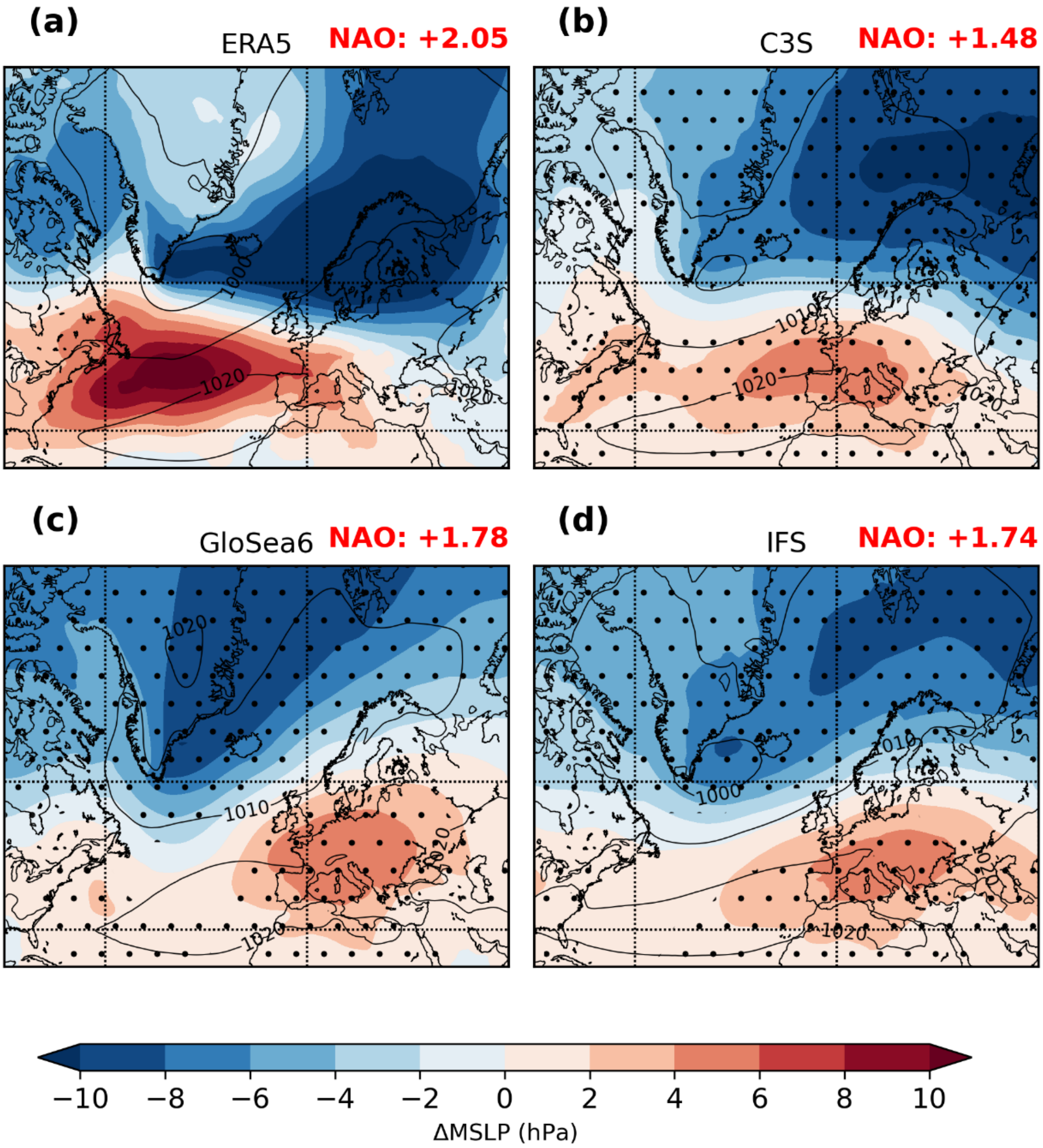

Fig. S2: February 2022 monthly mean MSLP anomaly due to the strong SPV. *February 2022 mean MSLP (hPa) for the North Atlantic region (20-90°N; 90°W-90°E) for* ***a)*** *ERA5 anomaly (shading) relative to climatology (1979-2021; contours).* ***b)*** *C3S anomaly between Strong and Average SPV members (shading) and ensemble-mean of Average SPV members (contours).* ***c)*** *same as b) but for GloSea6 and* ***d)*** *as in c) but for IFS. The monthly mean station-based NAO index anomaly (see Methods) is shown for each dataset. Stippling denotes statistical significance at the 95 % confidence level using a paired Student's t-test.*

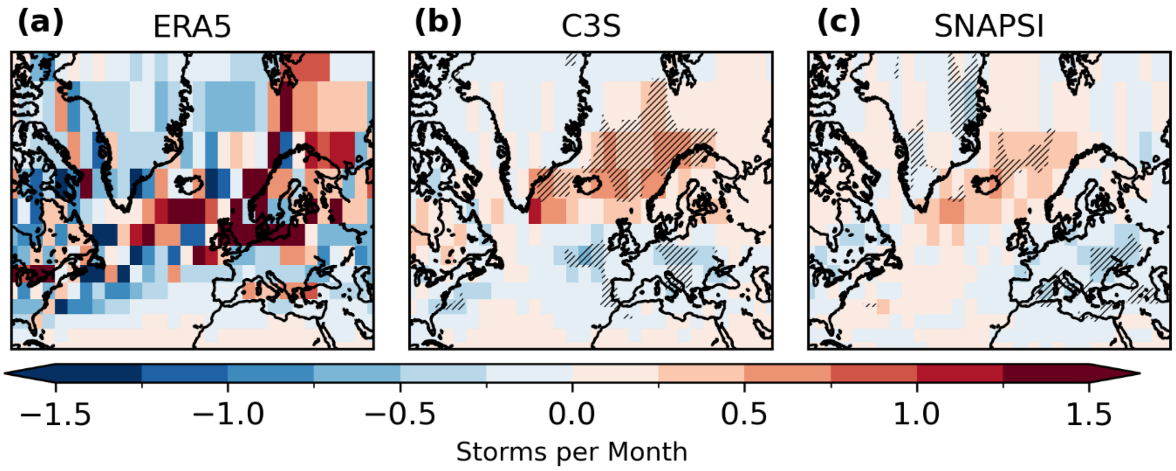

Fig. S3: Role of the strong SPV during February 2022 on cyclone density anomalies. *Monthly mean cyclone density anomalies aggregated in 5° x 5° bins for* ***(a)*** *ERA5 relative to the 1979-2021 February climatology;* ***(b)*** *C3S ensemble-mean Strong minus Average SPV forecasts, and* ***(c)*** *same as (b) but for SNAPSI (average of GloSea6 and IFS). Hatching denotes statistical significance at the 95% confidence level using a paired Student's t-test.*

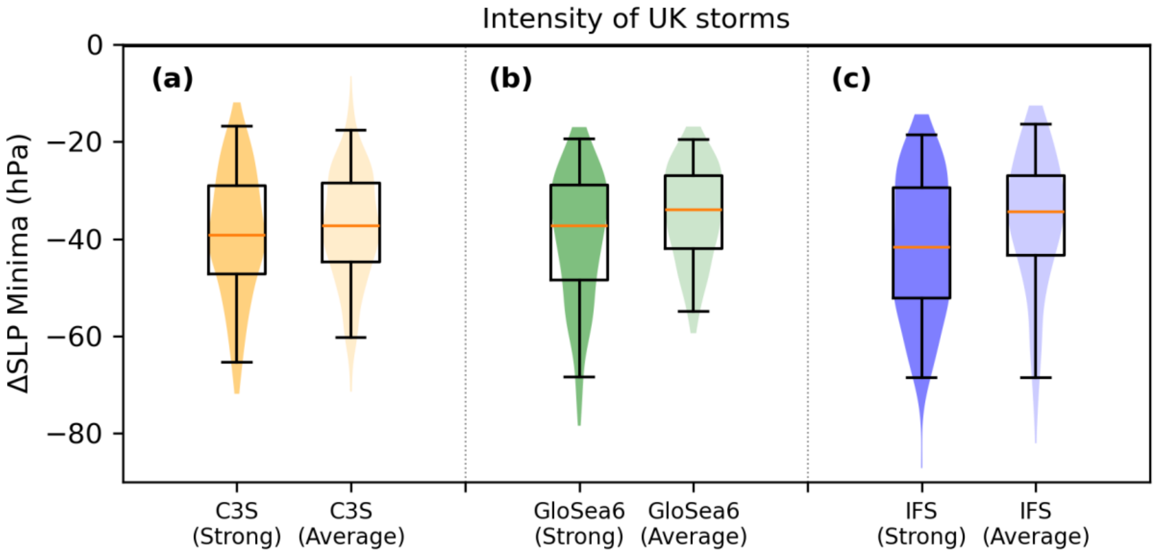

Fig. S4: Cyclone minimum central MSLP anomalies relative to climatology.

Maximum cyclone intensities (minimum central MSLP) over the UK region, expressed as along track pressure anomalies relative to the climatological background pressure for ***(a)*** *C3S Strong and Average SPV members,* ***(b)*** *same as (a) but for GloSea6 and* ***(c)*** *same as (b) but for IFS. Note that weak storms (p >990 hPa) are filtered out first, as in Figure 4. Whiskers show the 2.5th to 97.5th percentile range. The February monthly MSLP according to each respective reference dataset, which includes the ensemble-mean of the Average SPV forecasts, is removed along each cyclone track and the minimum MSLP anomaly is calculated. This removes differences in minimum MSLP that arise from a predominantly northward shifted storm track.*

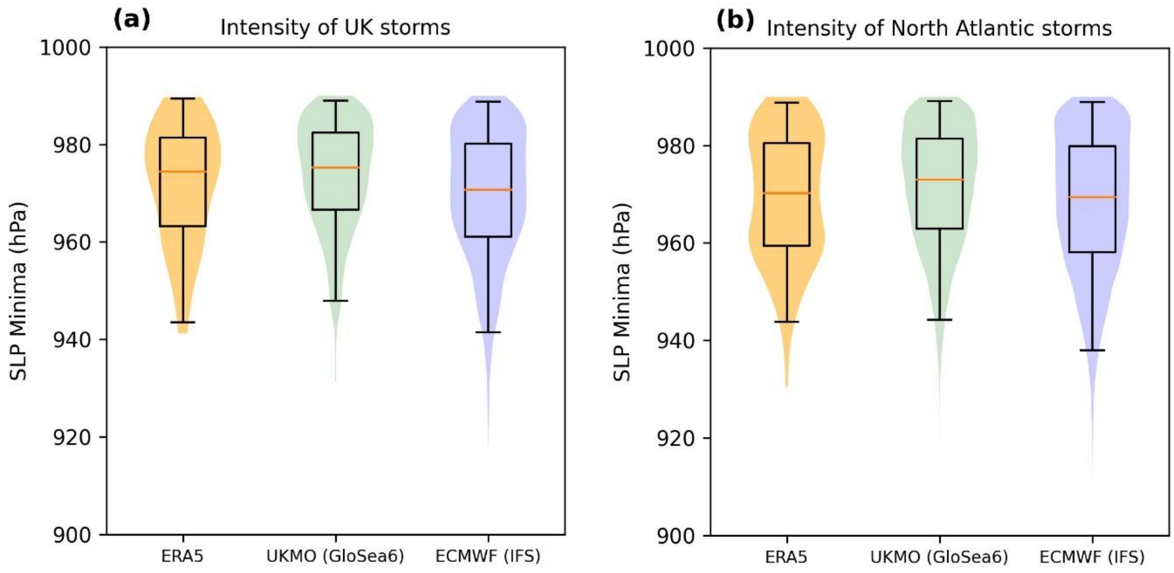

Fig. S5: Climatological evaluation of modelled cyclone intensity for the UK and North Atlantic regions during February. *Violin plots of maximum cyclone intensity during February in ERA5 (yellow), GloSea6 (green) and IFS (purple) hindcasts over a 23-year period (1994-2016) for* ***a)*** *the UK region (49.5-62.5°N; 20°W-4°E) and* ***b)*** *the North Atlantic region (50-70°N; 90°W-40°E).*

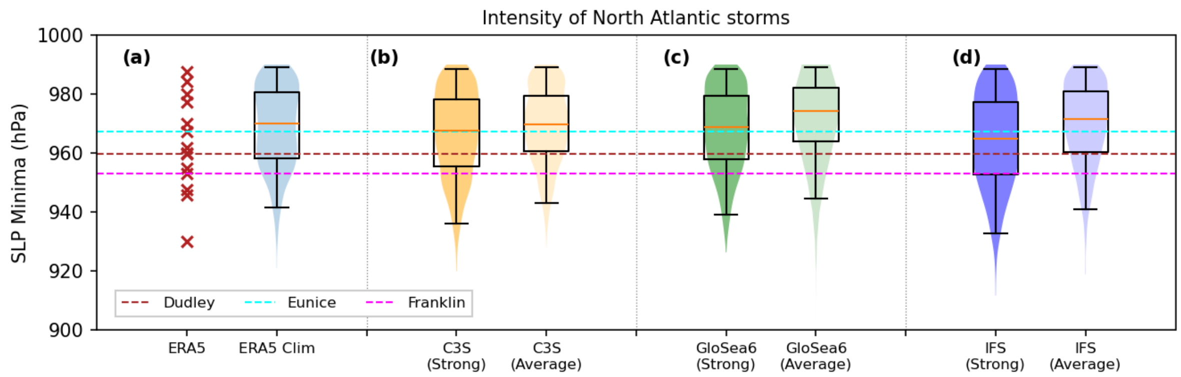

Fig. S6: Distributions of maximum cyclone intensity over the North Atlantic basin during February 2022. ***a)*** *Violin plots showing the distribution in ERA5 February 2022 versus 1979-2021 climatological minimum MSLP during storm lifetime within the North Atlantic domain (50-70°N; 90°W-40°E);* ***b)*** *same as (a) but for C3S Strong and Average SPV forecasts;* ***c)*** *same as (b) but for GloSea6 and* ***d)*** *same as (c) but for IFS. All storms with central MSLP minima above 990 hPa throughout the storm lifetime were excluded. Whiskers extend to the 2.5th and 97.5th percentiles of each distribution. Horizontal dashed lines correspond to the minimum pressure values of the three named storms (labelled) in February 2022.*

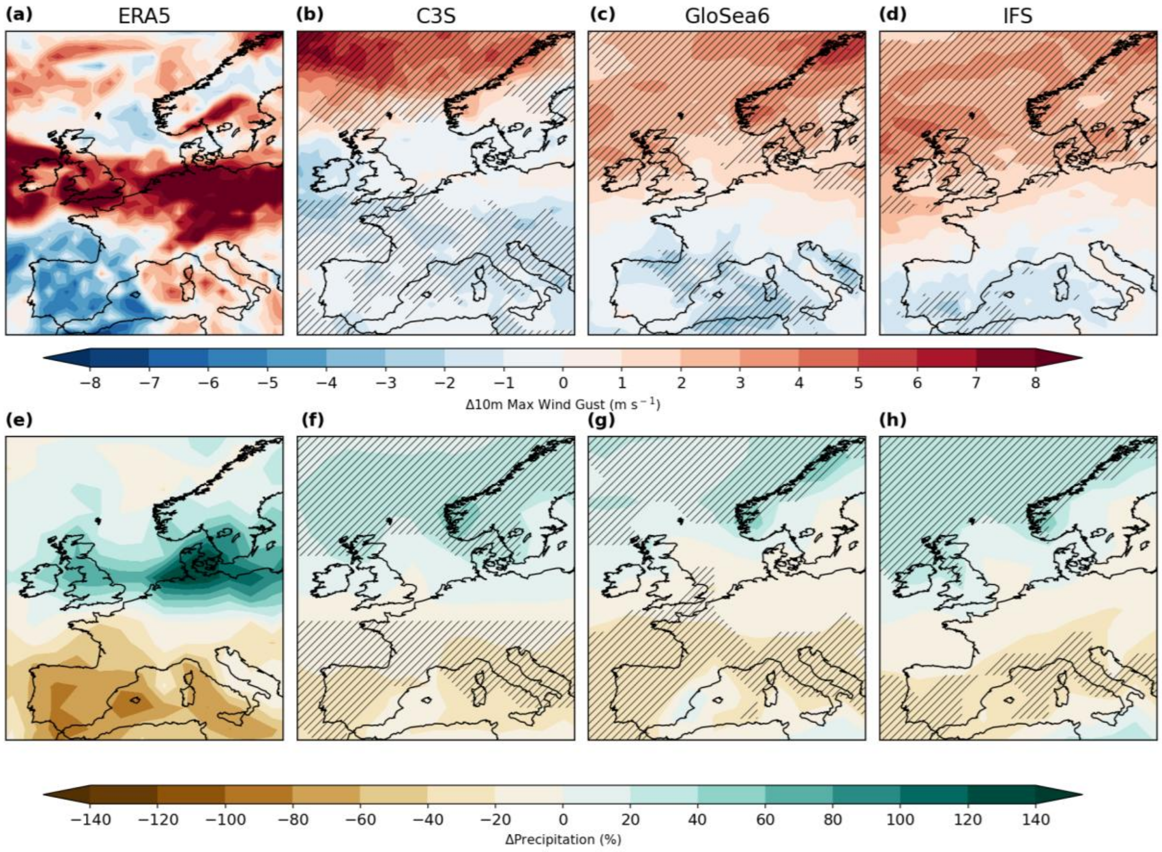

Fig. S7: Anomalous surface weather hazards during February 2022 due to the strong SPV. *February 2022* ***(a-d)*** *monthly maximum 10 m wind gust anomaly (m s^{-1}) and* ***(e-h)*** *monthly precipitation anomaly (%) for* ***(a,e)*** *reanalysis (ERA5 for wind gust and GPCP for precipitation),* ***(b,f)*** *C3S,* ***(c,g)*** *GloSea6 and* ***(d,h)*** *IFS. Observed anomalies are computed with respect to 1979-2021. For C3S, GloSea6 and IFS, anomalies are the ensemble-mean difference between the Strong and Average SPV forecasts. Hatching denotes statistical significance at the 95% confidence level according to a paired Student's t-test.*

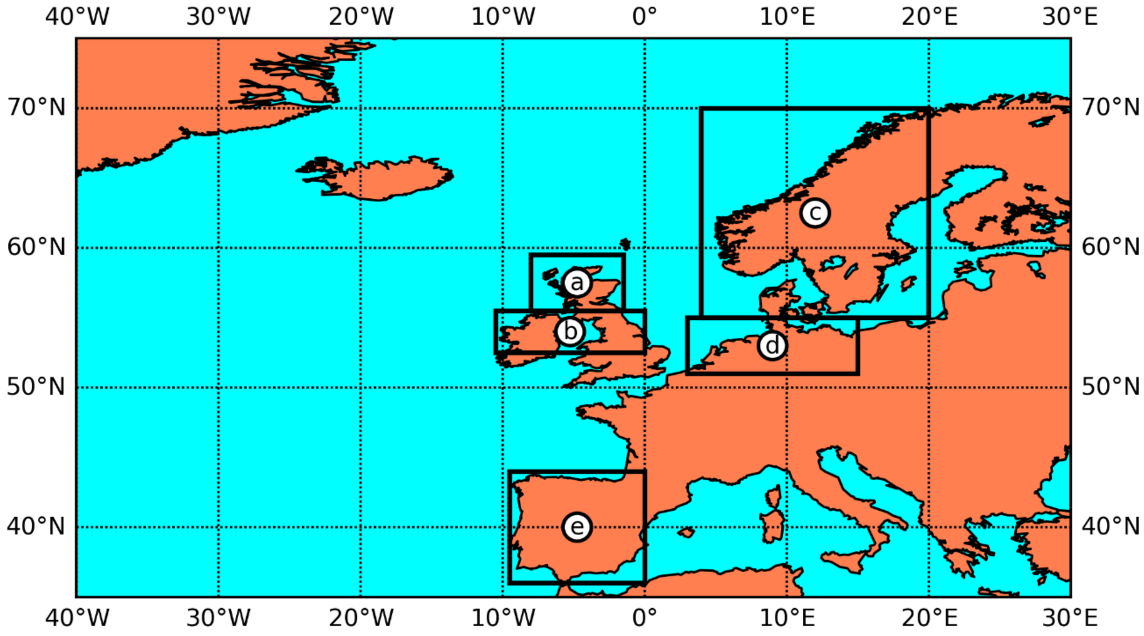

Fig. S8: Map of select regions across Europe to highlight regions with largest sensitivity to SPV influence. ***(a)*** *Scotland (55.5-59.5˚N; 8-1.5˚W);* ***(b)*** *Northern England and Ireland (52.5-55.5˚N; 10.5-0˚W);* ***(c)*** *Scandinavia (55-70˚N; 4-20˚E);* ***(d)*** *Northern Germany and the Netherlands (51-55˚N; 3-15˚E) and* ***(e)*** *Iberia (36-44˚N; 9.5˚W-0˚E).*

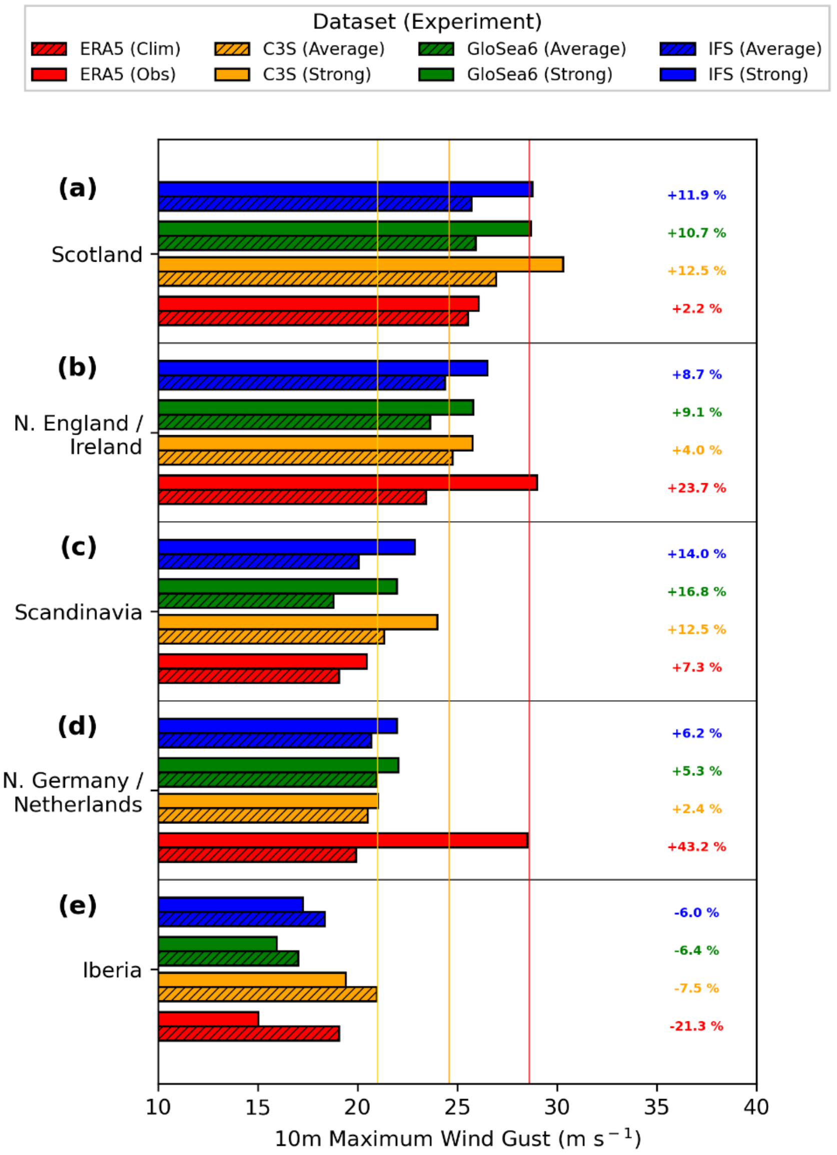

Fig. S9: Effect of the strong SPV on extreme 10 m wind gusts for different European regions. *February monthly maximum 10 m wind gust (m s^{-1}) computed over all grid cells (land area only) for* ***(a)*** *Scotland,* ***(b)*** *Northern England and Ireland,* ***(c)*** *Scandinavia,* ***(d)*** *Northern Germany and the Netherlands and* ***(e)*** *Iberia. C3S, GloSea6 and IFS show ensemble-mean values. Hatched bars show climatology for ERA5 and Average SPV conditions for C3S, GloSea6 and IFS. Unfilled bars denote February 2022 for ERA5 and Strong SPV conditions for C3S, GloSea6 and IFS. Vertical lines denote strong gale (yellow), equivalent to Beaufort Force 9/~21 m s^{-1} (47 mph); whole gale (orange), equivalent to Beaufort Force 10/~25 m s^{-1} (55 mph); and violent storm (red), equivalent to Beaufort Force 11/~29 m s^{-1} (64 mph). The percentage difference in experiments with a strong SPV is labelled (far right).*

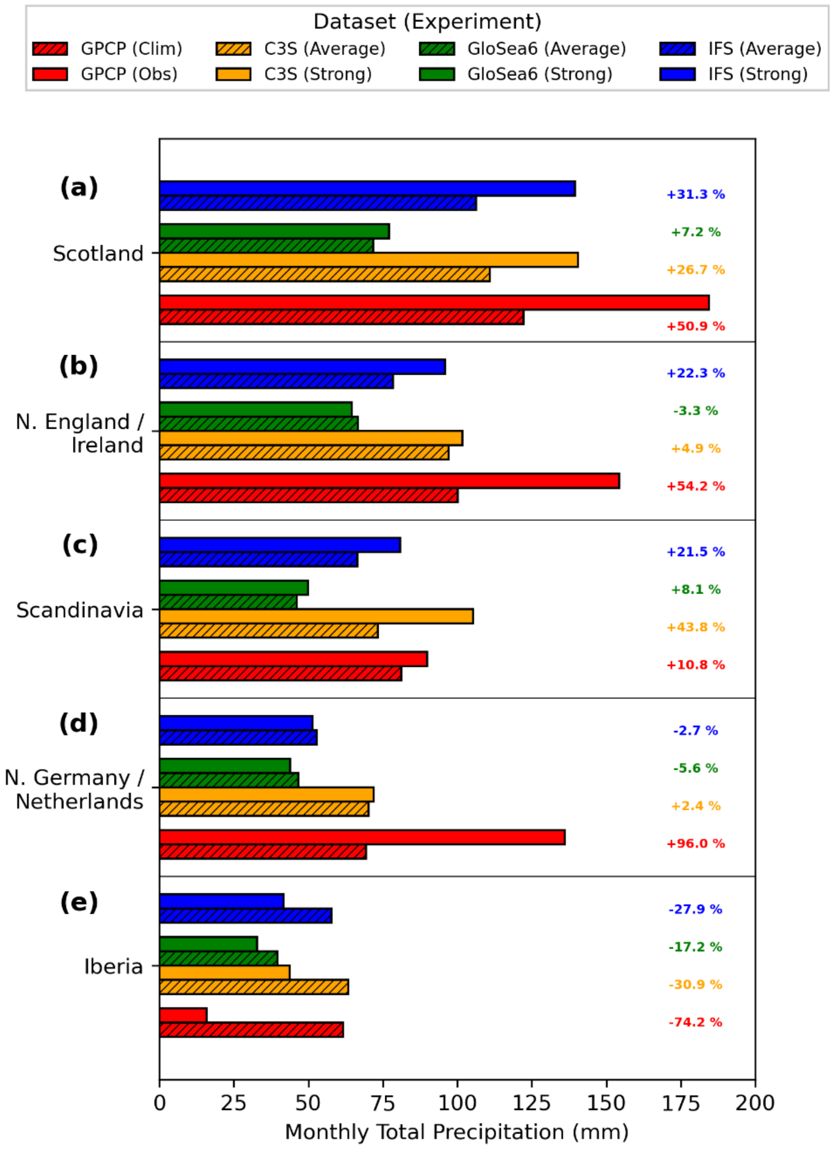

Fig. S10: Effect of the strong SPV on monthly total precipitation for different European regions. *February 2022 total precipitation (mm) computed over all grid cells (land area only) for* ***(a)*** *Scotland,* ***(b)*** *Northern England and Ireland,* ***(c)*** *Scandinavia,* ***(d)*** *Northern Germany and the Netherlands and* ***(e)*** *Iberia. C3S, GloSea6 and IFS show ensemble-mean values. Hatched bars show climatology for ERA5 and Average SPV conditions for C3S, GloSea6 and IFS. Unfilled bars denote February 2022 for ERA5 and Strong SPV conditions for C3S, GloSea6 and IFS. The percentage for a Strong versus Average SPV is labelled (far right).*

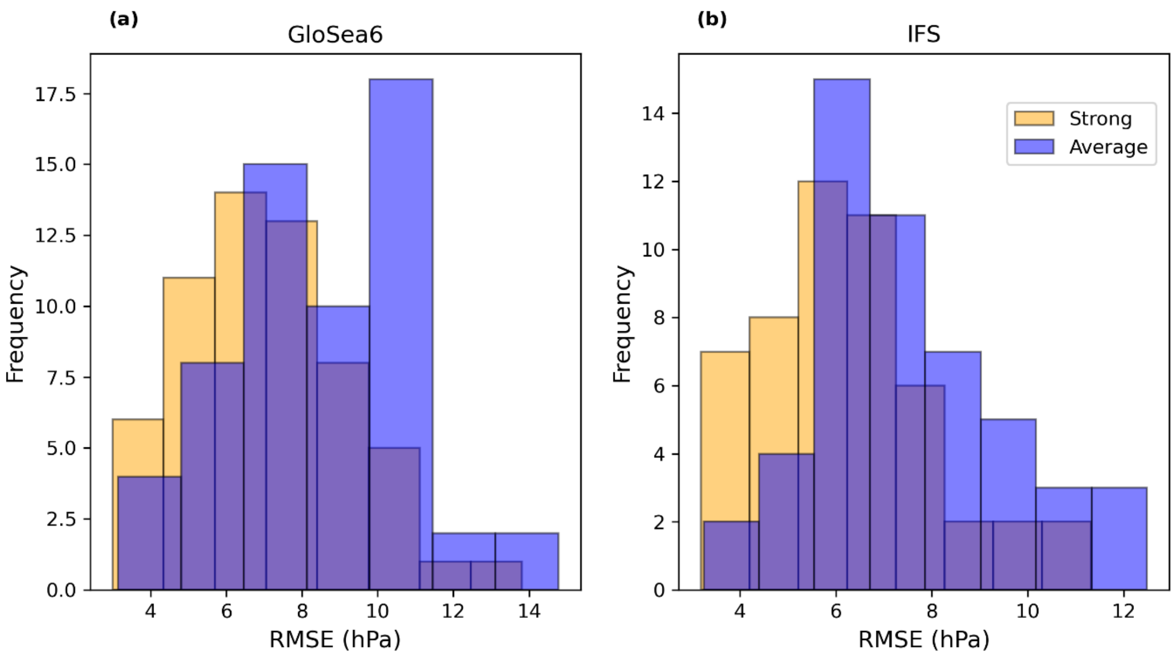

Fig. S11: Enhanced forecast skill for February 2022 North Atlantic monthly mean MSLP when accounting for the strong SPV state. *North Atlantic (30-90°N; 90°W-40°E) average root mean square error (RMSE) in February 2022 MSLP.* ***(a)*** *GloSea6 and* ***(b)*** *IFS for Strong (yellow) and Average (blue) SPV conditions. Histograms show RMSE across individual ensemble members.*

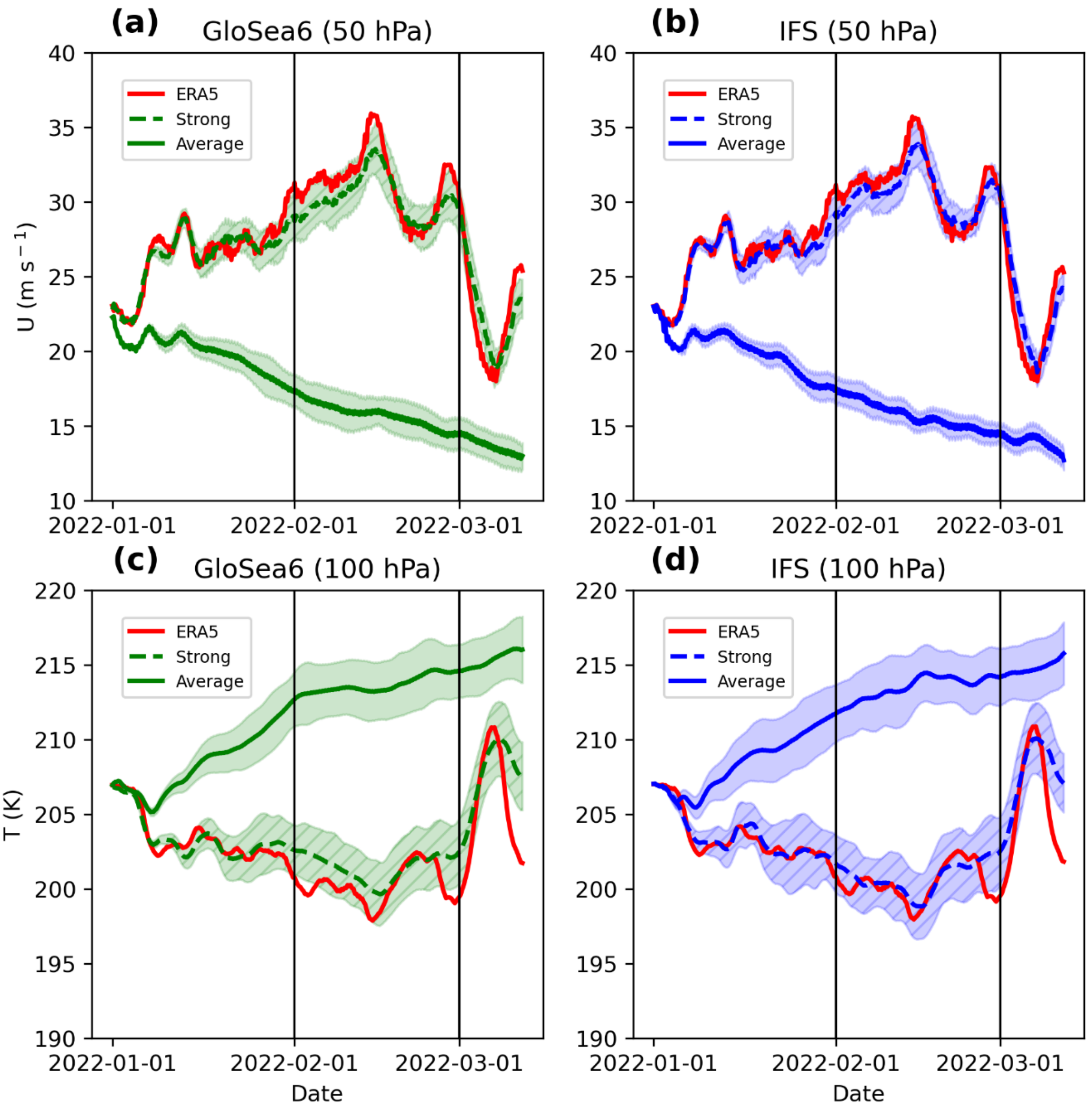

Fig. S12: Polar vortex nudging performance in GloSea6 and IFS. *Evolution of 50-70°N average daily zonal mean zonal wind ($\overline{U}$) at 50 hPa, according to ERA5 (red solid line) and the ensemble-mean of the respective Strong (dashed lines) and Average (solid lines) SPV state for* ***a)*** *GloSea6 (green) and* ***b)*** *IFS (blue).* ***c)*** *and* ***d)*** *as in a) and b), but for 100 hPa daily zonal mean polar-cap (70-90°N) temperature ($\overline{T}$). The shaded regions denote ±1 standard deviation of the ensemble spread (hatched for the Strong SPV state). Vertical lines mark the start and end of February 2022.*

Table S1: Strong SPV impact on distribution of UK cyclone intensity anomalies. *Cyclone minimum MSLP anomaly statistics for the UK domain during February 2022 for C3S, GloSea6 and IFS Strong and Average SPV conditions. To account for the effect of a shifted storm track on cyclone minimum MSLP, anomalies along tracks are computed by subtracting the background monthly mean MSLP from the reference simulation. The differences can be compared with those in Table 1, with the relative differences expressed as a percentage (values in parentheses).*

Dataset		Minimum cyclone pressure anomaly [hPa]				
		5th	25th	Median	75th	95th
C3S	Strong	-61.0	-47.2	-39.3	-29.1	-19.4
	Average	-58.9	-44.7	-37.4	-28.4	-21.4
	Strong – Average	-2.1 (68%)	-2.5 (119%)	-1.9 (107%)	-0.7 (175%)	+2.0 (-160%)
GloSea6	Strong	-62.7	-48.4	-37.3	-28.9	-21.3
	Average	-52.4	-41.9	-34.0	-27.0	-20.0
	Strong – Average	-10.3 (86%)	-6.5 (72%)	-3.3 (62%)	-1.9 (59%)	-1.3 (325%)
IFS	Strong	-65.0	-52.1	-41.7	-29.5	-20.2
	Average	-62.8	-43.4	-34.4	-27.0	-17.1
	Strong – Average	-2.2 (49%)	-8.7 (122%)	-7.3 (88%)	-2.5 (54%)	-3.1 (155%)

Table S2: Strong SPV impact on North Atlantic storm statistics. *Cyclone frequency and cyclone minimum MSLP statistics for the North Atlantic domain during February 2022 for ERA5 (1979-2021), C3S, GloSea6 and IFS Strong and Average SPV states.*

Dataset		Frequency (month^{-1})	Minimum cyclone pressure [hPa]				
			5th	25th	Median	75th	95th
ERA5	Climatology	14.1	944.7	958.2	969.9	980.7	988.3
C3S	Strong	14.0	940.9	955.3	967.7	978.2	987.4
	Average	13.0	946.4	960.5	969.8	979.4	988.0
	Strong – Average	+1.0	-5.5	-5.2	-2.1	-1.2	-0.6
GloSea6	Strong	14.4	943.5	957.9	968.6	979.5	987.9
	Average	12.9	949.7	964.0	974.3	982.2	988.8
	Strong – Average	+1.5	-6.2	-6.1	-5.7	-2.7	-0.9
IFS	Strong	15.1	937.8	952.7	964.9	977.1	986.8
	Average	14.2	944.7	960.2	971.4	980.8	988.4
	Strong – Average	+0.9	-6.9	-7.5	-6.5	-3.7	-1.6

Table S3: Relative risk change in damaging wind gust potential due to the Strong SPV. *February 2022 Storm Severity Index (SSI) (see Methods) for European sub-regions. Values shown are the ensemble-mean with parentheses giving the 95% confidence intervals based on bootstrap sampling with replacement. Significantly different risk ratio values between the Strong and Average SPV state are shown in bold and underlined (determined where the confidence intervals do not overlap). SSI values are integrated over the month based on the maximum within each non-overlapping 3-day interval (i.e., 1st to 4th February; 4th to 7th February etc., n=9). Values are computed over land only.*

Dataset		Scotland	N. Eng /Ireland	Scandinavia	N. Germ /Netherlands	Iberia
C3S	Strong	6.2 (1.7, 10.0)	2.6 (0.0, 4.6)	26.9 (9.1, 41.7)	1.0 (0.0, 1.7)	2.4 (0.6, 4.0)
C3S	Average	1.3 (0.4, 2.1)	1.0 (0.3, 1.7)	8.2 (2.0, 13.1)	0.6 (0.1, 1.0)	3.9 (0.6, 6.5)
C3S	Strong/ Average	4.77	2.60	3.28	1.66	0.62
GloSea6	Strong	0.4 (0.2, 0.5)	0.4 (0.2, 0.6)	9.3 (6.6, 11.7)	0.4 (0.0, 0.5)	0.9 (0.4, 1.3)
GloSea6	Average	0.1 (0.0, 0.1)	0.1 (0.1, 0.2)	3.0 (1.8, 4.0)	0.3 (0.2, 0.5)	0.9 (0.6, 1.2)
GloSea6	Strong/ Average	**<u>4.00</u>**	**<u>4.00</u>**	**<u>3.10</u>**	1.33	1.00
IFS	Strong	0.3 (0.2, 0.5)	0.6 (0.0, 1.0)	7.6 (5.4, 9.5)	1.5 (0.8, 2.1)	0.5 (0.2, 0.7)
IFS	Average	0.1 (0.0, 0.2)	0.3 (0.1, 0.4)	1.9 (1.2, 2.5)	0.5 (0.3, 0.7)	1.3 (0.5, 2.0)
IFS	Strong/ Average	**<u>3.00</u>**	2.00	**<u>4.00</u>**	**<u>3.00</u>**	0.38

Table S4: SNAPSI model UK and North Atlantic cyclone frequency and intensity biases.

Climatological cyclone frequency and cyclone minimum MSLP statistics for the UK and North Atlantic regions during February for GloSea6 and IFS. Data are based on hindcasts over a 23-year period (1994-2016).

<table>
<tr><th rowspan="2"></th><th rowspan="2">Dataset</th><th rowspan="2">Frequency (month^{-1})</th><th colspan="5">Minimum cyclone pressure [hPa]</th></tr>
<tr><th>5th</th><th>25th</th><th>Median</th><th>75th</th><th>95th</th></tr>
<tr><td rowspan="3">UK region</td><td>ERA5</td><td>3.4</td><td>950.9</td><td>963.3</td><td>974.5</td><td>981.4</td><td>987.9</td></tr>
<tr><td>GloSea6 – ERA5</td><td>-0.4</td><td>+1.1</td><td>+3.4</td><td>+0.7</td><td>+1.1</td><td>+0.3</td></tr>
<tr><td>IFS – ERA5</td><td>-0.2</td><td>-5.4</td><td>-2.2</td><td>-3.7</td><td>-1.2</td><td>-0.2</td></tr>
<tr><td rowspan="3">North Atlantic region</td><td>ERA5</td><td>15.1</td><td>947.5</td><td>959.4</td><td>970.2</td><td>980.5</td><td>988.2</td></tr>
<tr><td>GloSea6 – ERA5</td><td>-2.2</td><td>+1.4</td><td>+3.5</td><td>+2.8</td><td>+0.9</td><td>-0.0</td></tr>
<tr><td>IFS – ERA5</td><td>-0.8</td><td>-5.1</td><td>-1.3</td><td>-0.8</td><td>-0.6</td><td>-0.2</td></tr>
</table>
